# International Conference on Women and Infectious Diseases

**DOI:** 10.3201/eid1011.040612

**Published:** 2004-11

**Authors:** Marian McDonald, Martha Anker, Carolyn Deal, Alison Mawle, Siobhán O'Connor, Larisa Slaughter

**Affiliations:** * Centers for Disease Control and Prevention, Atlanta, Georgia, USA;; † World Health Organization, Geneva, Switzerland;; ‡ National Institutes of Health, Bethesda, Maryland, USA

**Keywords:** women, conference, infectious diseases, introduction

On February 27–28, 2004, scientists, clinicians, researchers, women's health advocates, educators, policymakers, and representatives from nongovernmental organizations and community-based organizations gathered in Atlanta for the first International Conference on Women and Infectious Diseases (ICWID): From Science to Action. The Office of Minority and Women's Health of the National Center for Infectious Diseases, Centers for Disease Control and Prevention (CDC), spearheaded the conference. It was cosponsored by the World Health Organization, the Pan American Health Organization, the Department of Health and Human Services (DHHS), and the American Society for Microbiology.

The broad ICWID steering committee included representatives from the DHHS Office on Women's Health, the National Institutes of Health Office for Research on Women's Health, the National Institute of Allergies and Infectious Diseases, the National Institute for Child Health and Human Development, the Fogarty Center, and the Office of Women's Health of the Health Resources and Services Administration. Academic, community-based, and philanthropic organizations as well as numerous CDC entities were involved. The conference's goal was to enhance prevention and control of infectious diseases among women worldwide. The conference's 400 attendees from 25 countries (both industrialized and developing nations) and 30 U.S. states recognized the need for a forum to address the complex set of concerns and issues surrounding women and infectious diseases.

Julie Gerberding, director of CDC and administrator of the Agency for Toxic Substances and Disease Registry, opened the conference with an overview of the impact of infectious diseases on women. The address reminded listeners of the female face of infectious diseases: women may be biologically more susceptible to certain infections and suffer more severe complications. Other keynote speakers included Paul DeLay of the Joint United Nations Programme on HIV/AIDS, Carol Bellamy of the United Nations Children's Fund, and Mirta Roses Periago of the Pan American Health Organization.

The theme of the conference was "From Science to Action." In 35 sessions, attendees discussed practical application of scientific knowledge in areas such as HIV, infectious causes of chronic diseases and other infectious disease–chronic disease relationships, gender roles in infectious disease transmission and prevention, sexual coercion and its effect on infectious diseases in women, sexually transmitted diseases, health disparities, healthcare workers and caregivers, immunization, effective community-based strategies, the role of cultural competence in women's health, and more.

The Bill and Melinda Gates Foundation sponsored 26 ICWID scholarships, which allowed persons from nongovernmental organizations and community-based organizations from 10 countries and four continents to attend who otherwise would not have had the opportunity. These ICWID scholars will amplify the conference's impact by taking the knowledge and insights gained back to their home countries and organizations.

The conference successfully illuminated the female face of infectious diseases. While celebrating successes in the prevention and control of prenatal and neonatal Group B *Streptococcus* infections and achievements in other arenas, participants emphasized the many challenges remaining for the future. With the continuation of such efforts, the newly spotlighted female face of infectious diseases can also be the face of hope and progress.

**Figure 1:**
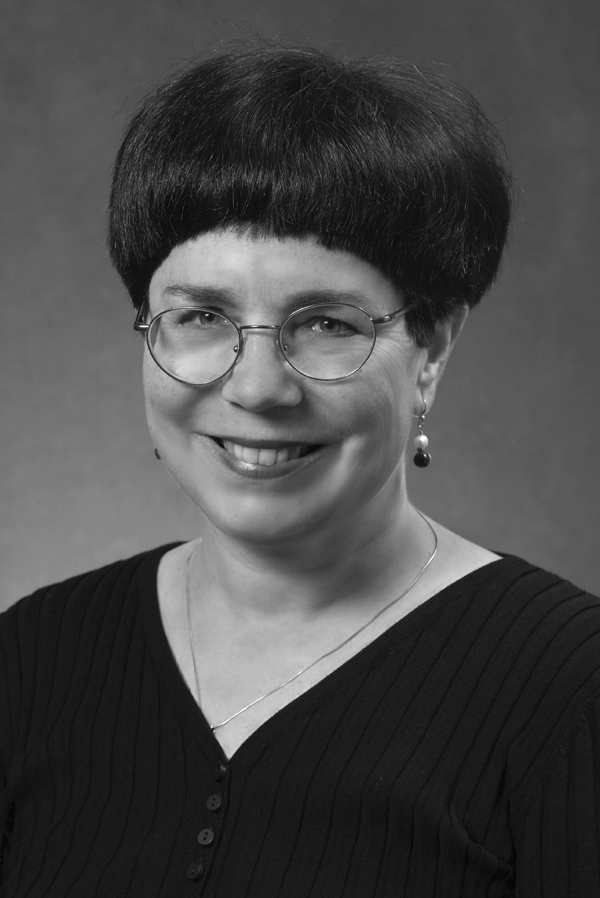
Dr. Marian McDonald serves as Associate Director for Minority and Women’s Health for the National Center for Infectious Diseases, CDC. She chaired the International Conference on Women and Infectious Disease: From Science to Action, held in Atlanta February 27–28, 2004. She has worked in women’s health and minority health for 3 decades.

**Figure 2:**
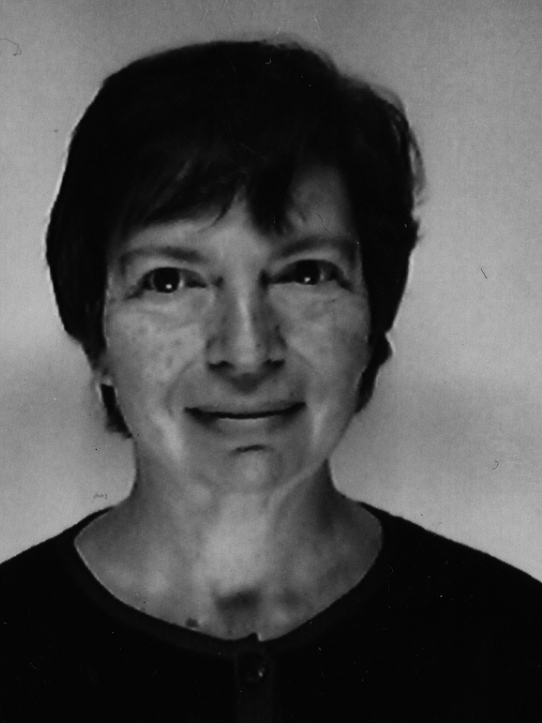
Ms. Anker has just retired from 25 years at the World Health Organization but continues to be active in research. She has researched gender issues throughout her career, most recently working on the effects of sex and gender on disease transmission and control during outbreaks of epidemic-prone infectious diseases.

**Figure 3:**
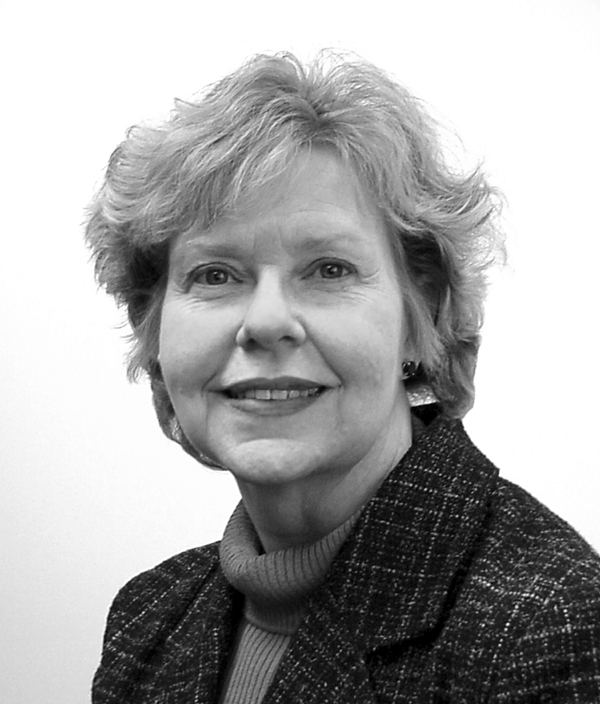
Dr. Deal is Chief of the Sexually Transmitted Infections Branch, Division of Microbiology and Infectious Diseases at the National Institute of Allergy and Infectious Diseases, National Institutes of Health. She has worked at Walter Reed Army Institute of Research as a Research Microbiologist, the Office of Vaccines Research and Review, and the U.S. Food and Drug Administration.

**Figure 4:**
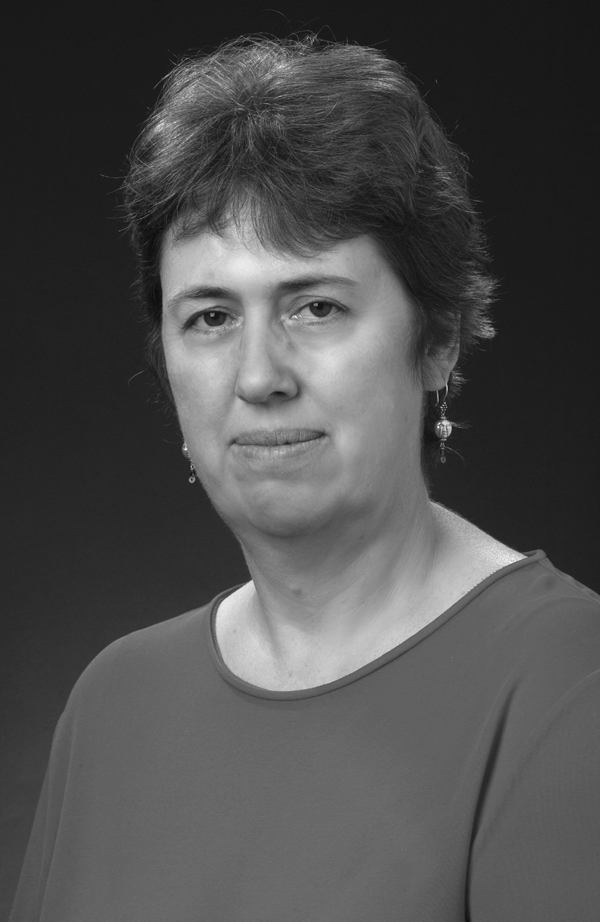
Dr. Mawle serves as vaccine coordinator for the National Center for Infectious Diseases, CDC. She began her career at CDC more than 20 years ago as an immunologist in the fledgling AIDS immunology laboratory.

**Figure 5:**
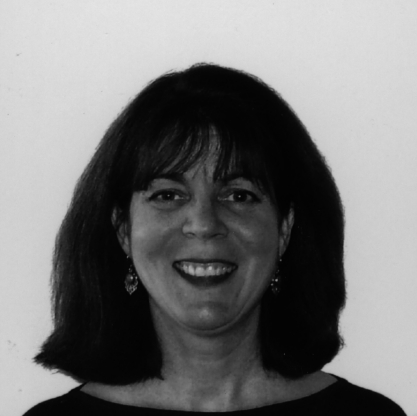
Dr. O’Connor serves as assistant to the director for Infectious Causes of Chronic Diseases, National Center for Infectious Diseases, CDC. She is active in developing the research agenda for and a strategic approach to this cross-cutting area, with known and potentially unrecognized impact on women.

